# Effective Treatment of Glioblastoma Multiforme With Oncolytic Virotherapy: A Case-Series

**DOI:** 10.3389/fonc.2020.00702

**Published:** 2020-05-14

**Authors:** Benjamin Gesundheit, Eliel Ben-David, Yehudit Posen, Ronald Ellis, Guido Wollmann, E. Marion Schneider, Karl Aigner, Lars Brauns, Thomas Nesselhut, Ingrid Ackva, Christine Weisslein, Arno Thaller

**Affiliations:** ^1^Rapo Yerape Ltd., Jerusalem, Israel; ^2^Department of Radiology, Shaare Zedek Medical Center, Jerusalem, Israel; ^3^Institute of Virology, Medical University of Innsbruck, Innsbruck, Austria; ^4^Christian Doppler Laboratory for Viral Immunotherapy of Cancer, Innsbruck, Austria; ^5^Division of Experimental Anesthesiology, University Hospital Ulm, Ulm, Germany; ^6^Medias Klinik, Burghausen, Germany; ^7^Arztpraxis am Regerplatz, Munich, Germany; ^8^Institut für Tumor-Therapie, Duderstadt, Germany; ^9^Praxisklinik fuer Allgemeinmedizin, Markt Berolzheim, Germany

**Keywords:** glioblastoma, immunotherapy, oncolytic virotherapy, biological therapy, Newcastle disease virus (NDV)

## Abstract

Glioblastoma multiforme (GBM) remains an incurable condition, associated with a median survival time of 15 months with best standard of care and 5-year survival rate of <10%. We report on four GBM patients on combination treatment regimens that included oncolytic virus (OV) immunotherapy, who achieved clinical and radiological responses with long-term survival, thus far, of up to 14 years, and good quality of life. We discuss the radiological findings that provide new insights into this treatment, the scientific rationale of this innovative and promising therapy, and considerations for future research.

## Introduction

Glioblastoma multiforme (GBM) represents ~50% of adult primary malignant brain tumors, which occur at an annual incidence of 2–3 per 100,000 adults (1), and is the most common cause of death among patients with central nervous system tumors. The standard treatment regimen includes resection followed by radiation and chemotherapy with temozolomide (TMZ) (2). Notwithstanding this aggressive approach, the median life expectancy for GBM patients is only 15 months (3), with limited treatment response after recurrence (4); only 5–10% of patients live for more than 5 years (5). With such a dismal prognosis, the need for new therapeutic approaches for GBM is significant.

For over a century, there have been anecdotal reports describing the coincidence of various viral or bacterial infections with tumor remission among cancer patients (6). Oncolytic viruses (OVs) have been characterized and defined as preferentially replicating in tumor cells and inducing their death while sparing normal cells (7). In addition to the direct lytic effect of OVs on tumor cells, a strong virus-activated innate and adaptive immune response contributes to the overall therapeutic outcome. These responses can overcome immunosuppressive forces in the tumor microenvironment, ultimately shifting “cold” tumors to “hot” tumors (8). The release of tumor-associated antigens and induction of immunogenic cell death subsequently stimulate anti-tumor immune responses with potential for long-lasting tumor control (9). Some OVs also infect tumor-associated endothelial cells, resulting in breakdown of the tumor vasculature and subsequent necrosis of uninfected tumor cells (10). Tumor cell preference for OV propagation is based on oncogenic signaling pathways or defects in innate antivirus responses frequently seen in malignant cells (11, 12). Recent years have seen significant breakthroughs in OV engineering, which has generated OVs encoding proteins that enhance their tropism for tumor cells (13–15).

While the first OV-based immunotherapy (virotherapy) has gained US Food and Drug Administration (FDA) and European Medicines Agency (EMA) approval for treatment of melanoma (16), oncolytic virotherapy for other tumor types is at various stages of clinical testing (17). Over the past three decades, OVs from >15 families have been preclinically assessed as potential treatment modalities for glioblastoma (18). Among these, nine have been included in numerous clinical trials (19). Importantly, these studies confirmed the general safety of OV application for brain tumors, with serious adverse effects rarely occurring. Durable complete responses were shown in up to 20% of patients, and regulatory fast-track designation by the FDA has been awarded to DNX-2401, Toca511, and PVS-RIPO (19).

Although the initial response is geared toward antiviral defense, the OV-elicited immune activation plays a major role in the therapeutic outcome (20). Consequently, virotherapy has gained significant attention as a partner for other immunotherapeutic approaches, such as dendritic cell (DC) therapy, cancer vaccines, T-cell therapies and immune checkpoint inhibitors (CPI) (21–23). CPIs selectively target immune inhibitory signals that contribute to the immune suppressive tumor environment, and thereby reinvigorate anti-tumor T-cell responses. CPIs have been shown to be particularly effective in combating tumors that are hypermutated or with specific neoantigen signatures (24), including recurrent, multifocal biallelic mismatch repair deficiency (bMMRD)-associated GBM (25). Tumoral OV infection precipitates endogenous DC migration and activation, which elicit a shift toward antitumor immunity. DC-based immunotherapies have been proposed to synergize with OVs (21, 26).

This case series presents the clinical and radiological outcomes of four patients with histologically-confirmed GBM treated with experimental combination virotherapy regimens as compassionate treatment. Given the nature of this early clinical experience and significant socio-economic factors, different exploratory treatment regimens involving a range of generically available OV strains were used. These cases are instructive for documenting clinical and radiological responses to virotherapy as an important basis for developing standardized and improved protocols for future clinical research.

## Case Presentation

**Figure 1:**
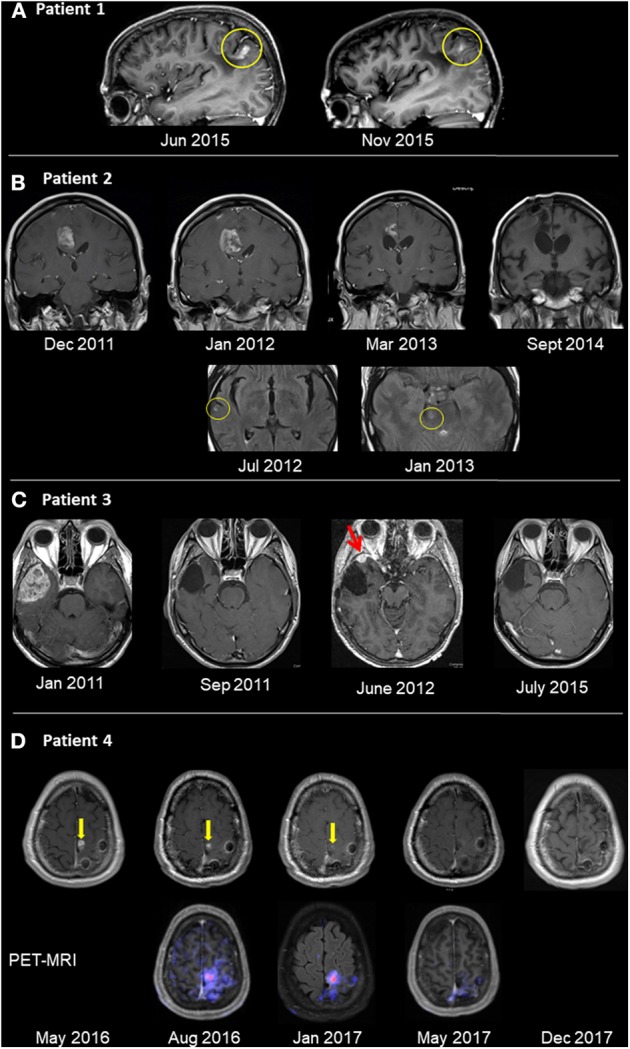
Radiological follow-up of GBM tumors treated with oncolytic virotherapy. **(A)** Patient 1 was diagnosed with GBM in 7/05 and underwent total surgical resection in 8/05. OV therapy was initiated in 5/06 and given for 3.5 years, after which patient remained with NED for 6.0 years. Recurrence in the left parietal lobe appeared on sagittal T1 contrast-enhanced images in 6/15 (left). The patient improved clinically after renewing OV alone. Five months thereafter, lesion shrinkage was observed (right). **(B)** Patient 2 was diagnosed with GBM in 10/10, underwent surgical resection and chemoradiotherapy. Relapse occurred (12/11) in the right frontal lobe (left image top row, T1 contrast enhanced images), with clinical deterioration. OV therapy was initiated. Follow-up imaging showed shrinkage until disappearance of the pathological enhancement. Concurrent to OV therapy and shrinkage, multiple scattered FLAIR hyperintense foci (with and without enhancement) were seen (bottom row, FLAIR images, yellow circle), possibly an immune-mediated response. **(C)** Patient 3 was diagnosed with a right temporal lesion (1/11; left image, T1 post contrast), underwent surgical resection and chemoradiotherapy, and no residual tumor mass was seen (9/11). Relapse occurred (6/12; red arrow), and following a second resection, OV therapy was initiated. The patient has no residual tumor (7/15; right image) and has remained with no radiological or clinical evidence of disease. **(D)** Patient 4 was diagnosed with GBM (10/15) and underwent resection and chemoradiotherapy. Following relapse in the surgical bed (5/16; yellow arrow, left image of T1 contrast enhanced images), OV was initiated. The focus of pathological enhancement decreased in size until disappearance (1/17). Concurrent PET-MRI images (bottom row) showed hypermetabolic activity even while Gd enhancement was decreasing.

Informed consent for publication was obtained from all patients in this case series. Regulatory approval for compassionate use was within the framework of the German Individueller Heilversuch. Patients were treated with individualized regimens comprised of three OVs: wild-type Newcastle disease virus (NDV) (Wageningen University, Netherlands), wild-type parvovirus (PV) (University Marburg, Germany), and wild-type vaccinia virus (VV) (Paul Ehrlich Institut Berlin, Germany). Each virus was used at a clinical dosage level of 10^9^ TCID_50_ (tissue culture infectious doses), as quantified by virus-specific cell-culture assays. Viruses were prepared for clinical use in conditioned cell culture medium that had been clarified by centrifugation, diluted to final TCID_50_ level as needed, sterile-filtered, stored frozen, and thawed on the day of injection. Viruses were injected to patients at intervals of ~2–3 weeks, administered by sequential 10 mL injections via the same catheter. A summary of the treatments and clinical and radiological presentations of the four patients is shown in Table 1 and Figure 1.

**Table 1:** Clinical summary of GBM patients undergoing OV therapy.

	**Patient 1**	**Patient 2**	**Patient 3**	**Patient 4**
Age (y) at diagnosis	33	54	43	46
Sex	F	F	M	M
MGMT hypermethylation	Negative	Positive	Negative	Negative
IDH1/2 status	Wild Type	n.a.	n.a.	Wild Type
Duration of Tx (y)	3.5 (break of 6 y) + 4.5	5.0	>7	>3.5
OS from Diagnosis	14.5 y (alive)	6 y (died 12 months after stopping OV)	8.5 y (alive)	>4 y (alive)
DC	+	–	+	–
Radiology	NED 10 y; relapse: PPG and shrinkage; SD 3 y	PPG and slow shrinkage = > CR; NED	NED since Tx	Resolution of residual disease → NED
Comments	Relapsed 6 y after Tx; PR after renewal of Tx	Discontinued Tx after 5 y; relapsed; died within 1 y.		NED since Tx

*CR, complete response; DC, dendritic cell therapy; NED, no evidence of disease; OS, overall survival; OV, oncolytic viruses; PPG, pseudo-progression; PR, partial response; SD, stable disease; Tx, treatment*.

### Patient 1

A previously healthy 33-year old woman presented in July 2005 (7/05) with slurred speech and left-arm weakness. CT studies showed a space-occupying lesion (SOL) in the left parietal lobe. Following total surgical resection (8/05), full neurological recovery was observed and corresponded with no radiological evidence of disease (NED) in the post-surgical MRI. Retrospective pathological evaluations revealed wild-type sequences for both isocitrate dehydrogenase 1 and 2 (IDH1/2) and O6-methylguanine-DNA methyltransferase (MGMT) promoter. Routine postoperative radiation (60 Gy) combined with oral TMZ (75 mg/m^2^/d for 42d) (12/05-1/06) was discontinued after 20 (instead of the usual 30) doses due to severe thrombocytopenia (6,000^*^10^9^/L) and extreme weakness (3/06). OV therapy was initiated (5/06) and administered via an intraarterial (IA) port system implanted end-to-side into the carotid artery. The patient remained stable with normal quality of life (QoL) and NED for 3.5 years after initiation treatment, which was discontinued due to pregnancy (10/09). After an uneventful pregnancy, she gave birth to a healthy baby. After a normal QoL for 6 years, she presented (6/15) with blurred vision, headache, stuttering, right hemiplegia, paranesthesia, hemianopsia, Jacksonian seizure, confusion, and disorientation in space and time. MRI (6/15; Figure 1A) confirmed relapse in the left parietal lesion measuring 9 × 7 × 15 mm. OV therapy was initiated (7/15) and resulted in prompt clinical improvement, including resolution of seizure activities and EEG findings of Jacksonian seizures. MRI (11/15) showed shrinkage of the tumor to 6 × 7 × 8 mm (Figure 1A). Virotherapy was continued with combinations of various viruses and switched (3/16) to IV administration due to complications with the IA port system. The patient has remained stable for 4.5 years after initiating the second phase of OV and 14.5 years after initiating OV treatment.

### Patient 2

A previously healthy 54-year-old woman presented with grand-mal seizures and left hemiplegia. She had gross surgical resection, and the tumor was found to be MGMT promoter methylation-positive. She received routine treatment (2/11) with radiation (60 Gy) and TMZ (75 mg/m^2^/d for 42 d, followed by 200 mg/m^2^ for 5 days/month). Full neurological recovery was observed. She relapsed radiologically, with an MRI (11/11) showing an irregular right frontal enhancing lesion (25 × 11 × 5 mm). The lesion did not respond to a second round of radio-chemotherapy, and clinically she progressed to left hemiplegia. There was no response to second-line chemotherapy with lomustine (110 mg/m^2^, Day 1/42) and procarbazine (60 mg/m^2^, Days 8–21/42), which was discontinued due to thrombocytopenia. The lesion grew rapidly (12/11; Figure 1B) to 29 × 18 × 25 mm and was accompanied by clinical deterioration. The patient then started IA OV treatment. Hemiplegia improved clinically 3 weeks after initiating OV therapy, although surveillance MRI 1 month after OV therapy (1/12; Figure 1B) showed an initial increase in tumor size to 30 × 32 × 30 mm, compatible with either true progression or pseudo-progression. However, follow-up MRI scans were compatible with central necrosis, followed by a slow decrease in the size of the enhancing lesion, until no radiological evidence of disease was seen (9/14; Figure 1B). Fluid-attenuated inversion, recovery (FLAIR) images captured during OV treatment showed multiple diffuse foci of FLAIR abnormalities (without clinical embolic causes, such as atrial fibrillation). For socio-economic reasons, the treatment protocol was modified to longer treatment intervals; the patient remained stable with NED. Five years from diagnosis, she decided to discontinue treatment for economic and psychological reasons. She then deteriorated clinically and radiologically and died 12 months later.

### Patient 3

A previously healthy 43-year-old man complained for 2 months of increasing headaches and weakness of his left leg. MRI (1/11) showed a SOL in the right temporal lobe (48 × 42 × 36 mm), which was surgically resected. Histology revealed MGMT-negative GBM. After standard chemo-radiation with TMZ (75 mg/m^2^/d for 42 d), he received five additional cycles of TMZ (200 mg/m^2^/d × 5 d/month) and recovered clinically. Follow-up MRI showed radiological relapse (13 × 10 × 10 mm) in the initial tumor bed (6/12; Figure 1C), and subsequently he underwent another surgery. Postoperatively, he refused further chemo-radiation and started OV treatment instead (8/12), which he continues to receive. The patient works, enjoys a normal QoL, and remains radiologically stable with NED (7/15; Figure 1C) for 8.5 years from initial diagnosis.

### Patient 4

A previously healthy 46-year-old male presented with right hemiparesis and unstable gait. MRI detected a left superior fronto-parietal SOL, which was surgically resected (10/15), resulting in clinical recovery. Pathology confirmed GBM with wild-type IDH. Despite negative MGMT, standard chemo-radiation with TMZ was given. Follow-up MRI showed suspicious findings of radiological relapse, with nodular enhancement, superior and inferior to the surgical bed (5/16; Figure 1D). Thus, the patient decided to proceed with monthly OV therapy. While remaining clinically asymptomatic, conventional MRI studies showed continued resolution of pathological enhancement over the next 12 months (8/16–12/17; Figure 1D). Interestingly, PET-MRI scans (8/16; 1/17; Figure 1D, bottom row) revealed diminishing gadolinium (Gd) enhancement and relatively stable hypermetabolic activity until disappearing during virotherapy (5/17). The patient remains clinically stable, conducts full physical work, and enjoys normal QoL >4 years after diagnosis.

## Discussion

The four presented GBM cases, all of which relapsed after standard treatments, benefited from treatment with various OV strains. The patients achieved complete response or stable disease, with long-term overall survival ranging between 4 and 14 years. Oncolytic virotherapy was well-tolerated and enabled improved QoL for years after its initiation. While the nature of this case presentation precludes a direct comparison to empiric median survival rates (5), the prolonged survival shows the potential of OV therapy for the management of GBM. These promising observations, corroborated by well-documented radiological responses, justify further development of standardized protocols for clinical trials in GBM patients. Combination therapy, integration of various types of OVs, and extended duration of treatment after full radiological response must be carefully considered, given that late relapse can occur (patients #1 and #2) despite NED in radiological surveillance. The choice of OV types, dosage and treatment frequency for each of the four patients was personalized, based on clinical responses to virotherapy (mainly body temperature, rash or clinical improvement) as well as socioeconomic factors. Despite these significant limitations of non-standardized treatment regimens and the very small number of patients, the radiological evidence and promising clinical outcomes illustrate the contribution of OV to GBM management, even at an advanced stage. As is the case of many protocols in oncology, different regimens can be clinically effective. Due to the heterogenicity of the four individual regimens administered, it is not yet possible to define the critical component(s) for future clinical study protocols. For example, regarding DC administration, patients #2 and #4 showed a good response without DCs, suggesting that DCs could be excluded from future protocols.

Monitoring tools remain to be established and optimized to quantify the “virogram,” tumor tissue OV sensitivity, viremia levels, circulating tumor cells and tumor biomarkers in the cerebrospinal fluid, immune responses to the OVs, and radiological observations monitoring responses to OV. Most importantly, monitoring specific immune profiles before and after OV immunotherapy might detect a modulation of the immune system indicative of activation of anti-tumor immune responses. Indeed, long-term survival among GBM patients has been correlated with improved immune status (27–29), which might explain the apparent success of OV immunotherapy in managing GBM.

At the time of initial diagnosis, brain micro-tumors are present. Thus, even advanced surgical techniques can provide only limited disease control, which might account for short-term extension of patient lifespan but with little impact on long-term survival. Current standard therapy leads ideally to minimal residual disease (MRD), which inevitably results in relapse. Consequently, future clinical trial designs should administer OV immunotherapies immediately following initial standard treatment with surgery and chemo-radiation, at which point patients are closest to MRD (30).

Future radiological protocols should place more focus on documenting unique immune responses such as those identified in the presented cases. Punctate foci with abnormal enhancement and FLAIR signals were seen in patient 2, both adjacent to as well as distant from the relapse site, a few months after initiation of OV therapy. MRI of patient 4 showed decreased pathological Gd enhancement following initiation of OV therapy, until it disappeared 12 months later. On the other hand, concurrent PET-MRI images showed hypermetabolic activity. Conventionally, these findings are interpreted as possible disease progression or alternative diagnoses (e.g., embolic stroke). However, since these foci disappeared over the subsequent few months, this phenomenon might be a pseudo-progression reflecting an inflammatory response to OV infection, as described for some immune therapies (29). Thus, optimal radiological surveillance is crucial for identification of the desired and unique immune responses to OV (31).

While mixtures of OV strains may convey additive clinical effects, systematic assessment in controlled clinical trials with multiple OVs remains challenging. To date, combinations of OVs in clinical trials have been limited to one oncolytic Maraba vesiculovirus component combined with an adenoviral vaccine vector (32). Preclinically, a few studies have addressed potential synergy of OV combinations, such as adenovirus and vaccinia (33), mumps and measles virus (34), and Newcastle disease virus (NDV) with reovirus and parvovirus (35). Furthermore, combination of multiple therapeutic modalities including IM with DCs and/or CPI, might provide superior results, and the combined or sequential use of various OVs might boost the therapeutic efficacy by avoiding neutralizing antibody responses elicited against a specific virus strain. Thus, it is important to monitor immune activation of patients undergoing OV therapy by testing for antibodies against original tumor tissue as well as OV-specific neutralizing antibodies. Moreover, activation of NK and T cells can be monitored and may be relevant in understanding states of clinical remission. Immune phenotypes may vary in individual patients when tested shortly after diagnosis and before surgery (36). Accordingly, we found individual variations, which may imply that sustained remission in GBM patients may be based on different effector cells. Ideally, each patient should be tested before and at different time points during OV therapy.

Engineered OV strains might further enhance direct tumor killing and tumor-specific immune activation (37). The feasibility of these options needs to be explored first in xenograft mouse models and syngeneic tumor models to validate the mode of therapeutic activity in controlled settings (38, 39). All of these considerations need to be investigated in phase 1 trials followed by randomized large multi-center, controlled clinical trials, with the ultimate aim of developing standardized clinical protocols to further improve the outcomes of GBM patients.

## Data Availability Statement

The datasets generated for this study are available on request to the corresponding author.

## Ethics Statement

Written informed consent for publication was obtained from all patients in this case series.

## Author Contributions

BG reviewed all data and prepared the manuscript. EB-D reviewed all radiological studies and prepared them for the manuscript. YP, RE GW, ES, LB, and TN contributed to the preparation of the manuscript and review of the literature. KA performed the surgeries. AT treated the patients with the help of IA and CW.

## Conflict of Interest

BG is the founder and CEO, RE is CTO and YP is researcher and medical writer of Rapo Yerape Ltd., KA is the director of the Medias Klinik for surgical oncology, and AT is the founder and director of the Praxisklinik für Allgemeinmedizin. The authors declare that this study received funding from Rapo Yerape Ltd., which had the following involvement in the study: analysis of data and preparation of the manuscript.
